# Understanding Outdoor Gyms in Public Open Spaces: A Systematic Review and Integrative Synthesis of Qualitative and Quantitative Evidence

**DOI:** 10.3390/ijerph15040590

**Published:** 2018-03-25

**Authors:** Janet Lok Chun Lee, Temmy Lee Ting Lo, Rainbow Tin Hung Ho

**Affiliations:** 1 Department of Social Work and Social Administration, The University of Hong Kong, Hong Kong, China; janet_lee@hku.hk (J.L.C.L.); temllt@connect.hku.hk (T.L.T.L.); 2 Centre on Behavioral Health, The University of Hong Kong, Hong Kong, China; 3 Sau Po Centre on Ageing, The University of Hong Kong, Hong Kong, China

**Keywords:** outdoor gym, environmental infrastructure, physical activity, public health

## Abstract

(1) Background: An outdoor gym (OG) is environmental infrastructure built in a public open space to promote structured physical activity. The provision of OGs is increasingly seen as an important strategy to realize public health agendas promoting habitual physical activity. A systematic review was conducted to synthesize characteristics of OG and OG users’ experiences and perceptions in different cultural contexts; (2) Methods: Online searches of multidisciplinary databases were conducted in health, sport and recreation, and urban planning disciplines. Characteristics of OGs were synthesized by integrating evidence from quantitative, qualitative, and mix-methods studies. The experiences and perceptions of OG users from both qualitative data and survey responses were synthesized through framework analysis; (3) Results: Nine studies met the inclusion criteria (three quantitative studies, four mixed-methods studies, and two pure qualitative studies). None were excluded on the basis of quality. OGs mainly serve adult and older adult population groups. Their size, design, and instructional support vary across studies. The inclusion of functional types of equipment did not have a unified standard. Regarding experiences and perceptions of OGs, five major themes emerged: “health”, “social connectedness”, “affordable”, “support”, and “design and promotion”; (4) Conclusions: The OG characteristics synthesis guides the direction in further studies regarding exploration of design parameters. The qualitative and quantitative synthesis revealed that health was a central theme of users’ experiences. OGs are also spaces where community-dwellers can find social connectedness while participating in structured physical activity at no cost. Findings from this review create knowledge support for OG as environmental infrastructure for further research and facilitate the understanding of users’ experiences and perceptions of OGs in different cultural contexts.

## 1. Introduction

The promotion of physical activity is an important public health agenda, as it is well documented that adequate physical activity is vital for disease prevention and health promotion [1]. Traditionally, physical activity interventions have focused on individual factors (i.e., self-efficacy) [2] and social factors (i.e., support from family and friends, affiliation groups) [3] to change behavior. Although these interventions have had some success in changing behavior, a growing amount of research shows that physical activity is affected by multiple factors (i.e., individual factors, social factors, and environmental factors) [4,5].

The social ecological model suggests that health behavior (e.g., uptake of and adherence to physical activity) is a result of inter-relationships between individuals and their social and physical environment [5]. Based on this model, research studies have investigated how the role of a neighborhood’s open spaces is related to its inhabitants’ health-related lifestyle choices [6] and how green space acts as a therapeutic landscape for seniors [7]. These research studies have focused on understanding how macro design in open spaces or natural landscapes provides opportunities for active lifestyle choices such as walking-for-transport and walking-for-leisure physical activity [8]. In addition to these studies, some researchers [9,10] have started to investigate the role of “outdoor gyms” (OGs) (physical activity infrastructure in open space) in influencing structured physical activity. 

An OG is environmental infrastructure in an open space or an area in a park that consists of a cluster of weather-resistant exercise machines or exercise stations that assist individuals to perform physical activity [11,12,13]. Some OGs were built to serve older adults [14,15]; some were designed to serve diverse population groups [10,16]; and some were built along with other recreation infrastructures (footpaths) to add diversity and intensity to users’ physical activity practice [17,18]. Despite these differences, the current literature supports the use of OGs as an important environmental strategy to realize a public health agenda of promoting physical activity [19]. Therefore, it is important to gain a more in-depth understanding of OG characteristics and users’ experiences of OGs and how users or open space visitors perceive this infrastructure. As health behaviors are largely shaped by contextual and cultural factors [20], qualitative data collected within a study participant’s daily environment might provide information from these perspectives. As far as we know, there has been no systematic review of mixed-methods studies concerning the current impact of OGs on individuals. Therefore, this review aims to (1) systematically synthesize the characteristics of different OGs documented in the included studies; and (2) synthesize recurring themes related to experiences and perceptions of OGs. An understanding of these issues could provide support to the more effective use of OGs in public open spaces and contribute to the development of intervention research on using OGs for promoting public health.

## 2. Methods

To provide a more comprehensive understanding of the research questions that this review sought to address, both qualitative and quantitative evidence, including qualitative, quantitative, and mixed-methods studies, was chosen to be included in the review. This type of review is useful when there is limited research on the topic being investigated. 

### 2.1. Search Strategy

**Figure 1:**
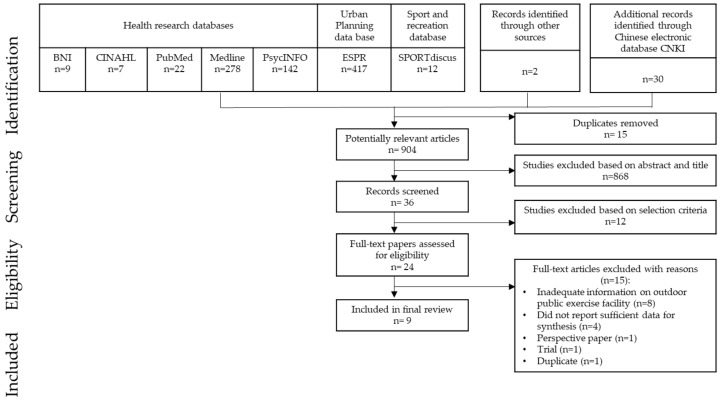
Flow chart of paper selection.

Online searches of the following English and Chinese multidisciplinary databases covering health, sport and recreation, and urban planning were conducted: British Nursing Index (BNI), Cumulative Index to Nursing and Allied Health Literature (CINAHL), PubMed, Medline, PsycINFO, SPORTdiscus, Environmental Science and Pollution Research (ESPR), and the China journals full-text database (CNKI). All potentially relevant literature, both published and unpublished, with no date restrictions, was searched. The bibliographies of the identified papers were examined for additional references. Field researchers were contacted to identify grey literature. The main search was completed in October 2017. A topic-oriented search approach was adopted. The search terms included a combination of key words related to OGs (e.g., park, outdoor gym, outdoor exercise equipment, stretch station, active park, geriatric park, elderly fitness corner, fitness zone, open gym). Search strategies were refined for each database. A back-and-forward tracking procedure was performed to identify additional relevant articles. Articles were first screened by abstract and title, 36 records were retrieved, and 12 studies were then excluded after closer examinations. A total of 24 articles were assessed for eligibility in full-text. Figure 1 presents a flow chart of the systematic literature search according to PRISMA guidelines.

### 2.2. Inclusion and Exclusion Criteria

Studies were included if they (1) Investigated OG as the main focus; (2) Explored the views and perceptions of OGs either quantitatively or qualitatively. Quantitative studies, qualitative studies, and mixed-methods studies were eligible for inclusion. Both peer-reviewed journal articles and grey research literature (conference papers, theses, letters to editors, brief reports) were eligible for inclusion. Studies were excluded if they (1) mainly explored physical activity experiences in green spaces or parks; or (2) contained insufficient data for synthesis. 

### 2.3. Data Extraction 

The study selection and data extraction followed a three-stage process: (1) database searching; (2) screening based on title and abstract using inclusion and exclusion criteria; and (3) selecting papers for review and data extraction. Two reviewers (Janet Lok Chun Lee (JLC), Temmy Lee Ting Lo (TLT)) were involved in the data selection and data extraction process to improve accuracy and avoid bias. Database searching, paper selection, and data extraction were conducted by JLC. Paper selection and data extraction were cross-validated by TLT. Data were extracted using a customized Excel spreadsheet. Extracted information included year of publication, sampling country, study aim, participant characteristics, sample size, study design, sampling method, data credibility method, analysis method, and results. Disagreements were resolved by consensus until full agreement was reached.

### 2.4. Quality Assessment

The criteria for quality assessment were based on the Critical Appraisal Skills Program checklist [21]. The checklist comprises 10 items that assist in appraising qualitative research systematically. As the checklist does not provide a scoring system, the current study applied a scoring system adopted by previous qualitative systematic reviews [22,23]. The first two questions were marked out of two (Yes = 2 and No = 1), and the remaining eight questions were marked out of three (Yes = 3; Somewhat = 2; No = 1). A maximum sore of 28 could be attained. Study quality was assessed by JLC and TLT. Interrater reliability was evaluated by Cohen’s *κ*, and a high level of agreement was reached (*κ* = 0.82). The quality of the quantitative studies was assessed using the STROBE (Strengthening the Reporting of Observational Studies in Epidemiology) checklist [24]. Rating of “high”, “medium”, or “low” quality was assessed by JLC and TLT. Consensus was reached in the case of discrepancies. This rating system was adopted by a previous mixed-methods systematic review [25]. No study was excluded on the basis of quality.

### 2.5. Data Synthesis

Synthesizing characteristics of OGs. Textual and numerical evidence of the characteristics of OGs from quantitative, qualitative, and mixed-methods studies were extracted and synthesized. 

Synthesizing experiences and perceptions of OGs. The experiences and perceptions on OGs from both qualitative data and survey responses were synthesized through framework analysis developed by Ritchie, Lewis [26]. Firstly, all of the findings from quantitative studies, themes, and informants’ quotes from qualitative and mixed-methods studies were extracted from the included studies. Then, the analysis underwent five stages: (1) Familiarization; (2) Identifying a thematic framework; (3) Indexing; (4) Charting; and (5) Mapping and Interpretation. The findings were read and re-read to establish familiarity, and a thematic framework was established with discussion with the second author. Participants’ quotes were indexed according to the thematic framework. Qualitative and quantitative data were then charted, mapped, and interpreted. Disagreements were resolved by discussion. 

## 3. Results

### 3.1. General Characteristics

Nine studies published between 2012 and 2017 were included in the systematic review (Table 1. The qualitative assessment score of the included qualitative and mixed-methods studies ranged from 18 to 22 (max possible score: 28). The quality ratings of quantitative studies were of low to medium level. Both published (i.e., peer-reviewed journal articles) and grey literature (i.e., theses) were included in this review. The studies were conducted in Australia, Canada, Brazil, Taiwan, China, USA, and Chile. Four studies were mixed-methods studies, two were pure qualitative studies, and three were quantitative studies. Four studies collected data from both facility users and neighborhood residents, and five studies collected data from facility users only. Among the qualitative studies and mixed-methods studies, five studies used face-to-face interviews and one study used open-ended survey questions to collect qualitative data. Most of these studies did not apply any qualitative research techniques to increase data credibility, and only two studies used peer debriefing to increase the trustworthiness of the data. 

### 3.2. Synthesis of Characteristics of Outdoor Gyms 

Table 2 addresses the first aim of this review, which was to synthesize the characteristics of different OGs documented in the included studies. Various terminologies, including “outdoor fitness equipment [29]”, “active park [27]”, “outdoor gym [11]”, “elderly fitness corner [28]”, “golden age gym [13]”, “third age fitness center [13]”, “national fitness path [30]”, “fitness zone [16]”, “open gym [31]”, “street gym [31]”, and “stretch station circuit [17]” were used in the studies to describe exercise equipment built in public open spaces. Most of the OGs investigated were located in public parks [13,16,17,27,28,29]; two were located in a non-specific public space [13,30], and one was located in public parks and squares [31]. The OGs described in half of the studies were targeted to be used or primarily used by older adults [13,28,29,30], and those described in the other studies were primarily used by young adults [31] and adults [11,16,17,27]. Half of the OGs had instructional support in the form of an instructor [13,30], exercise class [11,13], usage guide [11], and website [17] to assist in the usage of OG; the other OGs appeared to be unsupervised [16,27,28,29,31]. The number of equipment of OG ranged from five to 20 in the included studies, and two studies [17,30] did not clearly state the number of equipment in the study. In terms of the functional types of equipment, OG described in only one study [11] included the most comprehensive set of equipment/apparatus that targets aerobic, muscle-strengthening, muscle-flexibility, and balance with a purpose to assist individuals in meeting current physical activity guideline. Four studies [13,28,29,30] included the equipment name lists without explicitly providing details on the main function of the equipment. OG investigated in the rest of the studies either included equipment only for muscle-flexibility [17], only for musculoskeletal fitness [27], a combination of strength training and aerobic exercise [16], and a combination of muscle-strengthening, flexibility, and aerobic equipment [31]. Balance training equipment was not evident in almost all the studies [13,16,17,27,28,29,30,31].

### 3.3. Synthesis of Experiences and Perceptions of OGs

Table 3 provides an overview of the themes and subthemes in the reviewed articles. This addresses the second aim of this review, which was to identify recurring themes related to the experiences and perceptions of OG. The following five themes emerged from the data: (1) health; (2) social connectedness; (3) accessibility; (4) support; and (5) design and promotion.

#### 3.3.1. Health

The theme of health included the following subthemes: (1) rehabilitation; (2) physical health; (3) mental health; (4) fitness; (5) gain strength; (6) improve mood; (7) pursuit of health; (8) prevention; and (9) weight reduction.

The health theme was found in all of the reviewed studies. Participants expressed that using the equipment at OGs helped to treat health issues including frozen shoulder [29], post-surgery rehabilitation [29], spinal problems [27], and pain [28]. The participants in one study mentioned that they used OGs for weight reduction and as a form of leisure activity [30]. Users of OGs also perceived that they were improving their physical strength and general fitness [11], preventing disease, and maintaining their health [13,30]. The participants in two studies [28,29] mentioned that they “felt happier” after using the equipment.

Quantitative evidence showed 39% of the survey respondents of one study [16] indicated that losing weight was the most common reason for using the facility. Also, survey respondents of another study rated an average of 3.45 (SD = 1.59) on a seven-point Likert-scale on the statement “I feel fitter because I use this equipment” [17].

#### 3.3.2. Social Connectedness

The theme of social connectedness included the following subthemes: (1) new friendship; (2) benefits to family; (3) socialization; and (4) encouragement of other people.

Social connectedness was found in most studies. Participants expressed that users of OGs became friends and they liked the idea that they could exercise and also chat with their friends and get encouragement from others [13,28,29,30]. 

The subtheme of family was found in one study, in which a participant said she liked the idea that the OG allowed her to bring her children with her when she was doing exercise. She also saw it as a good role-modeling opportunity, as her children had a chance to see her being active [27]. 

#### 3.3.3. Affordable

The theme of affordable was found in two studies. OG is a free facility for the public. A participant in one study expressed that she and her family could not afford a paid gym and the OG was her only opportunity to receive resistance training [27]. The participants in one study [13] also mentioned that the fact that the OG was free-of-charge was a motivation to use it.

This theme was supported by survey data, in which survey respondents rated on average 3.77 (SD = 1.63) on a seven-point Likert-scale on the statement “I only do this type of exercise because the equipment is freely available” [17]. 

#### 3.3.4. Support

The support theme included the following subthemes: (1) maintenance and management; and (2) inadequate instrumental support.

Maintenance and management were recurring subthemes in most of the reviewed studies [27,28,29]. Participants expressed that when equipment in the OG was broken, it took a long time for it to be repaired. Participants mentioned that they had encountered irregularities with the equipment when they were using it. Some mentioned that they had not seen any management or maintenance of the OG since it was installed.

Participants in a few studies mentioned that they would like to have more instrumental support, such as having an instructor teaching them how to use the equipment, an exercise class with an instructor, or more instructions about the facilities [13,27,30]. 

#### 3.3.5. Design and Promotion

The design and promotion theme included the following subthemes: (1) quantity and variety of equipment; (2) safety; (3) advertisement; (4) shade; and (5) location close to attractions.

The quantity and variety of equipment were recurring subthemes. Participants were skeptical about whether the available circuits of equipment provided sufficient opportunities to exercise all parts of the body [30] and expressed that long waiting times were an issue [29].

Quantitative evidence showed that survey respondents of one study [17] rated on average 4.37 (SD = 1.47) on a seven-point Likert-scale for the statement “the [local government] should provide more equipment in the park”. 

The subthemes of shade and safety were related to facility design, while the subtheme of location close to attractions was related to landscape architecture design. One participant mentioned feeling uncomfortable exercising when there was strong sunlight [27]. 

Survey evidences showed that 87% of the survey respondents of one study [31] indicated that they combined the use of gyms with other types of exercise. Likewise, survey respondents of another study [17] rated on average 2.71 (SD = 1.29) on a seven-point Likert-scale for the statement “I come to this park specifically because of the stretching equipment”.

## 4. Discussion 

The current study systematically reviewed and integrated evidence from mixed-methods studies. Based on the inclusion criteria, nine studies were included in the review. The main objectives of the study were to support knowledge of OGs and identify recurring themes related to experiences and perceptions of OGs through qualitative and quantitative data synthesis.

An OG is important environmental infrastructure with the potential to influence a larger number of community dwellers to meet public health guidelines on physical activity in public settings [16]. The synthesis of characteristics of OGs of the included studies demonstrates that OGs were named in a variety of ways; in addition to the terminologies identified in this review, OGs are also called “exercise parks” [15], and “senior playgrounds” [32] in the literature. Unlike sport facilities, which have international standards for size and structure, the scale of OGs being investigated in the included studies ranged from having five pieces of equipment to 20 pieces of equipment. The number of equipment and the inclusion of functional types of equipment did not have a unified standard; whether or not there were the guiding principle behind the selection of the functional type of equipment is unclear. Only one study [11] included in this review mentioned that the selection of equipment pieces was “purposefully selected to meet the exercise needs of the broader adult population” in which equipment for aerobic fitness, upper and lower limb strength, balance, and flexibility were selected. According to information provided in the included studies, it seems that almost all OGs being investigated did not have equipment for balance training. Further research is needed for exploring the design parameter of OGs and this echoes with the calling of a recent perspective paper [33]. 

Synthesis of characteristics of OGs of the included studies shows that while some OGs in this review were primarily used by older adults [13,28,29,30], yet more than half of the OGs included in this review appeared to be unsupervised [16,27,28,29,31]; it appears that they operated like a children’s playground. Only some of the OGs were minimally supported by a user guide [11], occasional exercise class [11], supervision by a university student with a physical education background [13], and a physical education management instructor [30]. It seems that there is much room for improving the support provided to OG users, especially when some OGs are serving the older adults population group. The sub-group of this population might be frail and need more assistance in the form of instruction or education. 

In short, based on this synthesis, OGs of the included studies mainly serve adult and older adult groups. They are mostly under-supervised. The types of functional equipment available at OGs vary across studies and do not seem to have a unifying serving purpose or role. 

Regarding users’ experiences and perceptions of OGs, the theme of “health” was central to the experiences of OG users and residents in the neighborhoods in which OGs were located. This theme is supported by both qualitative and quantitative evidences. Participants used OGs for pursuing health, weight reduction, strengthening their physical health, and restoring health. Moreover, the health experience of using an OG was similar to the park visitation experience of the older population group. A quantitative park study that used a survey as a research instrument reported similar findings, with older park users ascribing their visits to parks as mostly health-related [34]. It is interesting to find that rehabilitation was a prominent subtheme under the theme of health. Participants in two studies reported experiences such as using OGs to treat their musculoskeletal disorders [27,29]. This subtheme, which emerged from participants’ quotes, provides some support for conducting future research that investigates the role of OGs in the healthcare system in self-managing long-term rehabilitation and health promotion.

The second most prominent and recurring theme regarding the experience of OG users was social connectedness. Social interaction with other users and the possibility of bringing other family members to the facility in a park environment were subthemes related to OG users’ experience. The theme of social connectedness provides preliminary grounds to establish further research on OG-based physical activity health promotion interventions, as a study that investigated the motivators for physical activity showed that people prefer interventions with a social component [35]. A previous qualitative study found that “group, peer and community support” and “meeting new people, forming new friendships and socializing” were important motivators for older adults adhering to physical activity habits [36].

Regarding perceptions of OGs, being affordable was a recurring attribute of OGs recognized by participants in the reviewed studies. This theme is also supported by quantitative evidence by a survey [17]. This finding is congruent with other qualitative findings of physical activity-related environmental research [8], and a qualitative review of physical activity interventions which found that affordability is an important factor for the success of physical activity interventions for older adults [36]. 

Similar to other research [37], which found “difficulty in use” to be a barrier to using a public exercise environment, the current review identified inadequate instructional support as a barrier to using OGs. Only four OGs [11,13,17,30] had some sort of instructional support for users. The finding of inadequate instructional support may provide a reason for current OG research demonstrating that users did not interact with the equipment with an adequate amount and intensity to achieve health benefits, although the usage rate of equipment was high [12]. 

The study participants’ perceptions of a lack of management and maintenance of OGs affecting usage is similar to the findings of previous environmental physical activity research [8]. The findings of this review contribute to the cumulative evidence [8,36,38] that management and maintenance is an important element that affects physical activity in public environments such as parks and other green spaces.

The quantity and variety of equipment was a prominent subtheme under the main theme “Design and Promotion.” Both qualitative and quantitative evidence is in support of the government providing a higher quantity of equipment in parks [17,29]. Apart from the quantity of equipment, the functional types of equipment varied across studies. If building an OG is seen as a potential public health strategy, this finding, together with the finding from the synthesis of characteristics of OGs, give support to the need for further research investigating the optimal quantity and functional types of equipment that will elicit the desired health outcomes and user satisfaction. This is important to consider as health benefits will only occur if an adequate dosage and type of physical activity is performed [1]. Lastly, both qualitative and quantitative evidence seems to suggest that OG users tend to perform other types of exercise besides just attending to OGs [17,27,31], so this finding gives support to park designers or urban planners that OGs should be built close to attractions or other exercise facilities. 

### 4.1. Limitations

To the best of our knowledge, this is the first study that integrates evidence from quantitative studies, qualitative studies, and mixed-methods studies on OGs in open environments. Several limitations should be acknowledged when interpreting the findings of this review. First, the studies included are not OG interventional studies, so information gathered for the OG characteristics synthesis might not be comprehensive enough. Second, non-published grey research literature was included. Non-published grey literature and most of the studies included in our review did not apply strategies that ensure data credibility (e.g., member checking, peer review, triangulation), and the quality of the review findings is thus compromised. Third, some studies included in this review were mixed-methods studies. Although these studies conducted interviews with neighborhood residents and facility users on their experience of or views on OG, they only reported the themes identified from the interviews by content analysis; supporting quotations were not reported, which made synthesis difficult and affected the comprehensiveness of the secondary data analysis. The findings of this review will not directly help with a specific decision in OG-related research, but this review adds to knowledge of this kind of infrastructure in public open space environments.

### 4.2. Strengths

Understanding OGs is an emerging and promising area of research. As this environmental intervention has the potential to affect population health, this review provides a valuable overview of evidence from disparate studies. OGs take slightly different forms in different countries. This may be the first review to attempt to synthesize the design, primary user group, and terminology used for OGs, and to give an overview of how users and neighborhood residents from different cultural backgrounds perceive this kind of physical activity–related environmental infrastructure. The current review included studies from both Asia (Taiwan, Korea, China) and Western countries (Australia, Canada, USA, and Brazil), which increases the generalizability of the findings. Both published and non-published research literature were included in the review, reducing publication bias. 

## 5. Conclusions

Synthesizing the design, primary-user group, and operation of OGs has created knowledge support for this kind of environmental infrastructure. Findings suggest that further studies on exploration of the design parameter and instructional support of OGs are needed. The qualitative and quantitative synthesis reveals that health is a central theme in users’ experience. OGs are also public spaces where community-dwellers can find social connectedness while performing structured physical activity at no-cost. The current review can guide the direction of further research into the functional roles of OGs in society. The results may also inform further interventional studies related to physical activity health promotion based on social-ecological theory. Further research should also consider and address the methodological limitations of the currently published findings. 

## Figures and Tables

**Table 1:** Study characteristics and methodological aspects of the included studies.

Study Characteristics	Number of Articles	Studies
Country		
Australia	2	[11,17]
Canada	1	[27]
Hong Kong	1	[28]
Taiwan	1	[29]
China	1	[30]
Brazil	1	[13]
USA	1	[16]
Chile	1	[31]
Participant characteristics		
Facility users only	4	[13,28,29,31]
Facility users and park users/neighborhood residents	5	[11,16,17,27,30]
Participants characteristics		
18–60+	6	[11,16,17,27,30,31]
>50	3	[13,28,29]
Type of publication		
Peer-reviewed journal (published)	7	[11,13,16,17,27,29,31]
Thesis (unpublished)	2	[28,30]
Methodology		
Pure qualitative	2	[13,29]
Mixed-methods	4	[11,27,28,30]
Quantitative	3	[16,17,31]
Qualitative data collection method (Qualitative studies and mixed-methods studies)		
Face-to-face interview	5	[13,27,28,29,30]
Open-ended questions	1	[11]
Data creditability (Qualitative studies and mixed-methods studies)		
Peer debriefing	2	[27,29]
Not reported	4	[11,13,28,30]

**Table 2:** Characteristics of outdoor gyms of the included studies.

Study	Country	Terminology; Location(s)	Supervision/Instructional Support	Primary User/Target User	Description of OGs; Equipment Name List (If Any)	No. of Equipment at Study Site	Type of Equipment #			
							Aerobic	Muscle-Strengthening	Muscle-Flexibility	Balance
[29]	Taiwan	“Outdoor fitness equipment”; Public parks	Not evident	Older adults	“Designs and shapes are similar to those found in gyms”; Arm stretch, shoulder wheel, air walker, waist twister, leg pliability developer, double surfboard, arm wheel, single bonny rider	6	√	Not clear	√	Not clear
[27]	Canada	“Active park”; Public parks	Not evident	Adults	“Parks with fitness equipment” “One active park 5 machines were clustered together...second active park had 5 separate stations, each with 3–4 machines and walking paths connecting them” “Equipment stations are freely accessible and can allow large numbers of people to engage in activities that improve musculoskeletal fitness”	5–20	Not clear	√	√	Not clear
[11]	Australia	“Outdoor gym”; Public parks	Instructional class & usage guide	Adults	“Contemporary mechanical installations consisting of a variety of robust fixed equipment targeting fitness, strength and balance may assist individuals in meeting current physical activity guidelines” “Equipment pieces were purposefully selected to meet the exercise needs of the broader adult population and also their suitability for older adults”	12	√	√	√	√
[28]	Hong Kong	“Elderly fitness corner’; Public parks	Not evident	Older adults	“aimed to provide suitable and free-of-charge equipment for elderly to stretch their muscles and improve level of coordination”; twister board, pedal bicycle, upper stretcher, polar circle and back support device	5	√	Not clear	√	Not clear
[13]	Brazil	“Golden age gym” “Third age fitness center”; Public parks and spaces	University student from physical education course as instructor; stretching and walking exercise program	Older adults	“These gyms are the result of a partnership between the municipality, a healthcare company, and the university” “Each has 10 exercise apparatuses”; Skiing, walking and horseback riding simulator, surf, stretcher, leg press, multi-exerciser, double diagonal rotation, vertical rotation, seated rowing	10	√	√	√	Not clear
[30]	China	“National fitness path”; Outdoor public spaces	Physical education management instructor	Older adults	“Built at outdoor, covered limited space; built according to landscape; simple and easily set up; small-scale investment; practical and appealing; convenient to the public; suitable for all ages; scientific, fun, fitness-oriented public exercise facilities”; Space walker, tai-chi wheel, twister, upper body stretch, stretching bar, trunk-strength machine, wheel for pairs, running machine, leg-press, gymnastic bars, back stretch, rider, shoulder rehabilitation machine, trunk massage for pairs	Not mentioned	√	√	√	Not clear
[16]	USA	“Fitness Zone”; Public parks	Not evident	Adult	“easy-to-use outdoor gyms consisting of durable, weather-, and vandal-resistant exercise equipment for strength training and aerobic exercise.”; “The equipment needs no electricity and is appropriate for individuals 13 years and older and for all fitness level.”	8	√	√	Not clear	Not clear
[31]	Chile	“Open Gym” “Street Gym”; Public parks and squares	Not evident	Young adult	“Typically composed of four to 12 different machines, each of which may contain more than one exercise unit, open gyms permit users to define and follow their own routines, as well as to execute them in the company of others.”	8–14 *	√	√	√	Not clear
[17]	Western Australia	“Stretch station circuit”; Public parks	Website	Adult	“installed at 500 metre intervals along one of the looping footpaths (2.5 km full circle);” “These stations were installed with the intent to promote physical activity, particularly in relatively inactive people…either be used simply to facilitate stretching; as a landmark to encourage interval training; or to gauge the exercisers’ effort and activity by virtue of the known distance between stations.”	Not mentioned	×	×	√	×

**#** Judged based on descriptions from text, equipment name, and equipment photos provided; * The 8–14 machines consist of 18–29 working units.

**Table 3:** Recurring themes, subthemes related to experiences, and perceptions of public exercise facilities.

Theme	Subthemes	Studies Supporting Subthemes	Supporting Quotations from Qualitative Data	Evidence from Survey Data
Health	Rehabilitation Physical health Mental health Fitness Gain strength Improve mood Pursuit of health Prevention Weight reduction	[11,13,27,28,29,30]	“I have a frozen shoulder problem, so I came to the park to do some arm stretches, and then, I came frequently to do the pull. Now, I feel that my shoulder is getting better and becoming more relaxed.” [29] “I had two discs in my back that were dislocated and I like to work out and stay in shape and keep my body strong and stuff. So I use it kind of as a therapy.” [27] “You will feel happier after using the equipment. It is good.” [29] “The air quality is good here and [I am] happier after having exercise.” [28]	39% of the fitness zone users reported that losing weight was the most common reason for using the fitness equipment (*n* = 345). [16] Survey respondents rated on average 3.45 (SD = 1.59) on a 7-point Likert-scale on the statement ‘I feel fitter because I use this equipment’(*n* = 182). [17]
Social connectedness	New friendship Benefits to family Socialization Encouragement of other people	[13,27,28,29,30]	“You come here frequently and you become familiar with the other people here; then, you become friends.” [29] “Very good, it seems like a family in this park … we become friends so we come every day.” [28] “Just a lot handier than actually going to the gym, I can bring my kid here and still get a workout in.” [27]	
Affordable	Free of charge	[13,27]	“I’m a low income parent. Going to the gym is not affordable for our family. That’s not an option … it’s my only option for resistance training equipment.” [27]	Survey respondents rated on average 3.77 (SD = 1.63) on a 7-point Likert-scale on the statement ‘I only do this type of exercise because the equipment is freely available’ (*n* = 180). [17]
Support	Maintenance and management Inadequate instrumental support	[27,28,29,30]	“The national fitness paths in our surroundings have not been receiving any management or maintenance since they were installed.” [30]“ … but we older adults don’t know how to use [this] equipment.” [29]	
Design and promotion	Quantity and variety of equipment Safety Advertisement Shade Location close to attractions	[11,27,29,30]	“I don’t think there are enough things to do there that people would [go] out particularly to do it. You don’t have anything like a basketball court or something that would draw people here for the exercise. If you were drawing people here for exercise and they wanted to spend a few minutes before or after doing something like that, it might be a bit different.” [27] “I have to take turns to use this equipment, and it is embarrassing to ask those using the equipment to give others a turn. Some people only sit on the equipment to rest, rather than exercise.” [29]	Survey respondents rated on average 4.37 (SD = 1.47) on a 7-point Likert-scale on the statement ‘the [local government] should provide more equipment in the park’ (*n* = 180). [17] 87% of the survey respondents combined the use of gyms with other types of exercise (*n* = 166). [31] Survey respondents rated on average 2.71 (SD = 1.29) on a 7-point Likert-scale on the statement ‘I come to this park specifically because of the stretching equipment’ (*n* = 183). [17]

## Supplementary Materials

The followings are available online at http://www.mdpi.com/1660-4601/15/4/590/s1, Table S1: Data Extraction, Table S2: Quality Assessment.
