# Supplementary material for: Understanding Outdoor Gyms in Public Open Spaces: A Systematic Review and Integrative Synthesis of Qualitative and Quantitative Evidence
Source: Int J Environ Res Public Health. 2018 Mar 25;15(4):590. doi: 10.3390/ijerph15040590 (PMC5923632; doi:10.3390/ijerph15040590)
Supplement: Supplementary file 1 [file ijerph-15-00590-s001.zip › Table S1. Data Extraction.pdf]

No.	Study	Year	Type	Country	Study Aim	Participant Characteristics	Age	n	Design	Data Crediibility	Sample Site	Result
1	Chow (2013)	2013	Peer-reviewed journal	Taiwan	Discover seniors' perception of, and needs for, OFE, thereby providing insight contributing to the planning and design of parks to improve park-based physical activity among older adults	User	50 to 97	55 Outdoor Fitness equipment user	Qualitative:<br>Semi-structured Interview (20 to 45 minutes)	peer debriefing double checked the content and accuracy of interviews	2 parks in Taiwan (Both with 6 pieces of equipments)	Themes<br>-Additional park feature to supplemnty walking or group activity<br>-Enjoyable natural environment<br>-Health Purpose/Rehabilitation<br>-Maintenance and management<br>-Developed new friendships from frequent visits<br>-Individualized style using the equipment
2	Copeland et al. (2017)	2017	Peer-reviewed journal	Canada	1) Examine how frequently active park equipment is used<br>2) Determine if active park fitness equipment may attract adults to park<br>3) Determine if PA intensity was higher among those who used the fitness equipment as compared to those who used parks in other ways<br>4) Garner community perceptions about active parks and ways to increase use	User and Non-user	18-60+	139	Mixed Methods:<br>Observation + Face-to-face interview (10 -15 minutes)	Osborne's suggestions establish trustworthiness of themes and subthemes	2 parks in Alberta<br>-one active park with 5 machines clustered together<br>-one had 5 separate stations, each with 3-4 machines and walking paths connecting them	-Theme 1: Health benefits<br>-Theme 2: Accessibility<br>-Theme 3: Benefits for families<br>Themes identified to increase park use<br>-Theme 4: Increase advertising within and outside the park<br>-Theme 5: Need for improved ground materials around the equipment<br>-Theme 6: Upkeep of the areas around the equipment to ensure proper drainage after rain<br>-Theme 7: Ensure adequate lighting<br>-Theme 8: Ensure the equipment remains remainn in good order<br>-Theme 9: Locate the equipment closer to other attractions such as playgrounds<br>-Theme 10: Improved instructions on the equipment and onsite trainers
3	Cranney et al. (2016)	2016	Peer-reviewed journal	Australia	1) Assess the impact of the installation and promotion of an outdoor gym in park setting on physical activity levels of park users<br>2) Examine the characteristics of outdoor gym users, motivators, enablers and barriers to use	User and Non-user	42.7	Post-installation:<br>534 Park User<br>219 Outdoor Gym User<br>Follow-up:<br>322 Outdoor Gym User<br>474 Park user	Mixed Model:<br>Before-after time series design with repeated observations and intercept interviews (Open-ended questions included motivators, enablers and barriers to outdoor gym use) Brief interview (5 minutes)	Not evident	Maroubra, Sydney	1) Most cited reason for using outdoor gym were to a) improve fitness (34%), and gain strength or tone muscle (26%)<br>2) Main enabler for outdoor gym use ited was for different types of equipment (25%), shade (15%) and more equipment (9%)<br>3) Most cited reason for not using the gym included lack of awareness of the outdoor gym (17%), a general dislike of or disinterest in the outdoor gym (15%), preference for a private gym (8%), no time (8%), not living close to the park (6%)
4	Choi et al. (2016)	2016	Thesis	Hong Kong	1) To assess the usage of elderly fitness corner at area of low socio-economic status<br>2) To explore the how elderly user perceive the facility	User	mean age=70	12	Mix-model:<br>Observation + Indepth Interview	Not evident	1 Park at area of low-socioeconomic status	Themes：<br>-Exercise at natural environment<br>-Weather as barrier<br>-Exercise revived the body, improved physical capability and joint movement<br>-Emotional well-being improvement<br>-The needs of maintenance and management for facility
5	Salin et al (2013)	2013	Peer-reviewed journal	Brazil	1) To describe the perception of older adults in terms of the motivation for entry and permanence in the Joinville AMI program	User	mean age = 67.22	163	Pure-qualitative<br>Semi-structured interview	Not evident	15 AMIs<br>city of Joinville, Santa Catarina	-Pursuit of health<br>-perceived benefits<br>importance of socialization, presence of instructors<br>-Prevention<br>-need because of a specific disease<br>-possibility of lesiure<br>-voluntarily<br>-free of charge<br>-encouragement of other people
6	Lee. (2015)	2015	Thesis	China	1) Usage; 2) Problems arised from using national fitness path<br>3) Provide recommendation	User and Non-user	18-75	495	Mix-model:<br>Literature review, survey, interview	Not reported	Chengde area countryside	'-強身健體，減肥，人際交流，娛樂休閒<br>(Strengthen Bodily Health, Weight-reduction, Social Interaction, Leisure pursuit)<br>-器材配備不充足<br>(Inadequate quipment)<br>-器材損壞<br>(broken equipment)<br>-體育鍛煉·意識不夠<br>(Inadequate knowledge in exercise)<br>-社會體育指導員配備不足<br>(Inadequate exercise instructor)
7	Cohen (2012)	2012	Peer-reviewed journal	USA	(a) how well is the TPL fitness equipment used after installation?;<br>(b) which age, gender,race/ethnicgroupsuseit, how often do they use it, and do they use it correctly?;<br>(c) overall,do more people use the parks? (Including both Fitness Zones and other park activity areas), and are park users more physically active in the parks than before the equipment was installed?	Observation: Park user and fitness zone user<br>Survey: Fitness zone user	15-60+	Interviewed 742 adult visitors at baseline, 942 at the first follow-up, and 952 at the second	Quantitative<br>Observational design + survey	Not applicable	12 Parks (48th street, Alondra, Athens, Cerritos, Gilbert, Ladera, Pathfinder, Salazar, Slauson, South, Steinmetz, Trinity)	-Peak usage time 9:30 - 11:30 and 3:30-5:30;<br>-Fitness Zone respondents did not differ demographically from the other park users interviewed: 80% Latino; 9%African; American; 61% female; averageage = 40 years, sd=12.5, p=0.94;<br>-Most common reason for using the fitness equipment is losing weight
8	Mora (2012)	2012	Peer-reviewed journal	Chile	Presents the results of a systematic observation and a survey of four open gyms	Observation: User<br>Survey: User	15-78	166 (survey)	Quantitative<br>Observational design + survey	Not applicable	4 Open gyms<br>(PdeValdivia, San Ignacio, Tobalaba, Canal San Carlos)	-Majority of the user is yound adult<br>-little difference between males and females in the four days of the observation, for women represented between 45% and 51% of users each day<br>-At the weekend, the proportion of elders reached 14% and the young adults represented 46%. On weekdays, the opposite was the rule, as young adults represented 59% of people and elders only 3%.<br>-87% combinedtheuse ofgymswith other types of exercise
9	Sibson et al. (2013)	2017	Peer-reviewed journal	Western Australia	Examine people's awareness, use, perceptions and attitudes in relation to specific type of outdoor exercise equipment	Park visitors	18 - 65+	400 (Survey)	Quantitative:<br>Self-completed questionnaire	Not applicable	1 urban park in Perth, Western Australia	'-Primarily used by adult<br>-Park user who usually exercise at vigorous level of intensity were more likely than those that exercise at low level of intensity to agree with the statement 'This equipment has increased my use of park'<br>-Stretch station user indicate that they 'enjoy using the equipment' (mean=4.16) (7-point likert scale)<br>-Stretch station user rated on average 2.71 on a 7 point likert scale on 'I come to the park specifically because of the stretching equipment'<br>-Stretch station user rated on average 3.45 on a 7-point likert scale on the statement ' I feel fitter because I use this equipment'<br>-Stretch station user rated on average 3.77 on a 7-point likert-scale on the statement 'I only do this type of eercise becasue the equipment is freely available'<br>-Stretch station user rated on average 4.37 on a 7-point likert scale on the statment ' the local [government] should provide more equipment in the park'
